# Behavioral, electrophysiological and neuropathological characteristics of the occurrence of hypertension in pregnant rats

**DOI:** 10.1038/s41598-019-40969-w

**Published:** 2019-03-11

**Authors:** Leandro F. Oliveira, Daniel J. L. L. Pinheiro, Laís D. Rodrigues, Selvin Z. Reyes-Garcia, Erika E. Nishi, Milene S. Ormanji, Jean Faber, Esper A. Cavalheiro

**Affiliations:** 1 Department of Neurology and Neurosurgery, UNIFESP/EPM, São Paulo, Brazil; 2 Department of Physiology, UNIFESP/EPM, São Paulo, Brazil; 3 Department of Nephrology, UNIFESP/EPM, São Paulo, Brazil; 4 Department of morphological science, Faculty of Medical Sciences, National Autonomous University of Honduras, Tegucigalpa, Honduras; 5 Centro Nacional de Pesquisa em Energia e Materiais, (CNPEM) – 13083-970 Campinas, SP, Brazil

## Abstract

Pre-eclampsia (PE) affects approximately 2 to 8% of pregnant women, causing blood pressure above 140 × 90 mmHg and proteinuria, normally after the 20th gestation week. If unsuccessfully treated, PE can lead to self-limited seizures (Eclampsia) that could eventually result in death of the mother and her fetus. The present study reports an experimental model of preeclampsia hypertension in pregnant (HP) and non-pregnant (H) Wistar rats by partially clamping one of their renal arteries. Pregnant (P) and non-pregnant (C) controls were provided. Differently from controls (C and P), H and HP animals presented a steady rise in BP two weeks after renal artery clamping. Injection of pentylenetetrazol (PTZ) induced behavioral and electroencephalographic seizures in all groups, which were increased in number, duration, amplitude and power accompanied by decreased latency in HP animals (p < 0.05). Consistent results were obtained in *in vitro* experimentation. Immunohistochemistry of hippocampus tissue in HP animals showed decreased density of neurons nuclei in CA1, CA3 and Hilus and increased density of astrocytes in CA1, CA3 and gyrus (p < 0.05). The present findings show that the clamping of one renal arteries to 0.15 mm and PTZ administration were able to induce signs similar to human PE in pregnant *Wistar* rats.

## Introduction

The increase in blood pressure during pregnancy, without known etiology characterizes gestational hypertensive syndrome (GHS)^1^. Preeclampsia (PE), a pathologic condition frequently occurring in late pregnancy characterized by edema, proteinuria and hypertension, is one of the hypertensive conditions classified as gestational hypertension syndromes^1^. It affects about 2 to 8% of pregnant women^2^ and epidemiological studies showed that approximately 76,000 pregnant women and 500,000 fetuses die due to PE annually^2^. Risk factors for PE include first gestation of a partner, multiple gestation pregnancy and pregnancy after 35 years old^3^. Risk factors involving comorbidity include chronic hypertension, chronic kidney diseases, diabetes mellitus and systemic lupus erythematosus^4^.

Preeclampsia is biochemically characterized by urinary protein levels equal to or above 300 mg/24 h and blood pressure (BP) equal to or above 140/90 mmHg with subsequent continual rise from a point around the 20^th^ gestational week^5–7^. The fetus growth and amount of amniotic fluid may be reduced as compared with normal gestation^8^.

A number of particular findings are presents in preeclamptic condition, such as, decreased levels of vascular endothelial growth factor (VEGF) and placental growth factor (PLGF), and increased levels of tumor necrosis factor (TNF-α), interleukin beta1 (IL-1b) and soluble fms-like tyrosine quinase-1 (sFlt-1)^9–11^.

The risk of preeclampsia involves its progression to eclampsia, a self-limited seizure condition that can lead to coma and death of the pregnant woman and her fetus^12–14^.

Medical management of PE is fundamentally directed towards the critical conditions with emphasis on blood pressure control and seizure suppression^15^, which leaves a narrow margin, if any, to try alternative methods or widening the understanding of this pregnancy disorder. Therefore, establishing a relevant animal model of preeclampsia could greatly help by shedding light on the mechanisms that can lead to PE.

Several PE models are suggested to mimic the major characteristic of preeclamptic condition, either by: (a) infusing either lipopolysaccharide^16^ (LPS) or interleukin beta 6^17^ (IL-6), or administering TNF-α^18^. The first is an inflammation-causing toxin whereas the two others are preeclampsia findings that denounce ongoing inflammatory process; (b) reducing blood flow to the placenta, as hypertension and reduced placental perfusion have been reported in preeclamptic condition; (c) infusing the anti-angiogenic factor, soluble fms-like tyrosine (sFlt-1), a variant protein present in the serum of preeclamptic women; (d) working on genetically engineered animals that develop hypertension^19,20^.

Some of these models require effort and time, while others involve a high degree of variability. But more importantly, they do not convincingly reproduce the key features of preeclampsia simultaneously and consistently, i.e., high blood pressure and low threshold for sustained convulsions.

Here we present an alternative possibility of producing preeclamptic findings – high blood pressure, proteinuria, increased TNF-α and reduced PLGF levels in the serum, reduced weight of offspring at birth, and increased seizure susceptibility *in vivo* and *in vitro* – achieved through the effects of reduced blood supply to the left (for convenience) kidney by means of the clipping of the left renal artery of female *Wistar* rats.

## Results

### Blood pressure and biochemical markers

**Figure 1:**
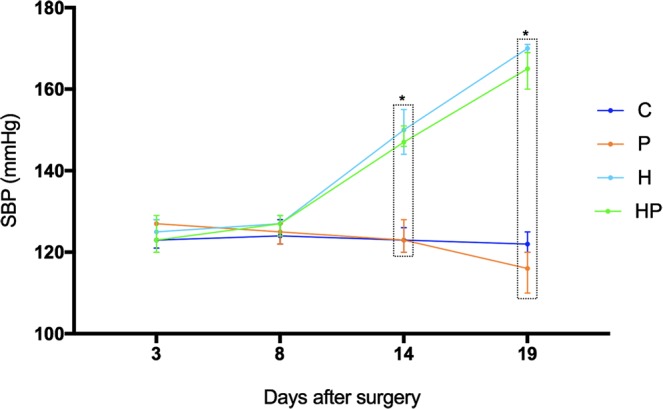
Evolution of systolic blood pressure (SBP). Mean values in control (C, dark blue), pregnant (P, light brown), hypertensive (H, light blue) and pregnant-hypertensive (HP, green) animals. Notice the progressive increase in SBP in H and HP groups. Levels indicated on the graph showing (*) were found to have statistically significant difference. Statistics details in Supplementary Material Fig. 12.

The mean values of systolic blood pressure (SBP) were similar in all groups (C, P, H and HP) when assessed on day 3 or 8 after surgery (F = 1.736; p = 0.169; F = 0.254; p = 0.858, respectively). SBP on days 14 and 19, however, was significantly higher in animals submitted to clamping of the left renal artery (H and HP groups) when compared to those of the groups C and P (H = 27.63; p < 0.0001 and H = 30.12; p < 0.0001, respectively). SBP levels in these groups remained high until the end of experiments (Fig. 1).

**Figure 2:**
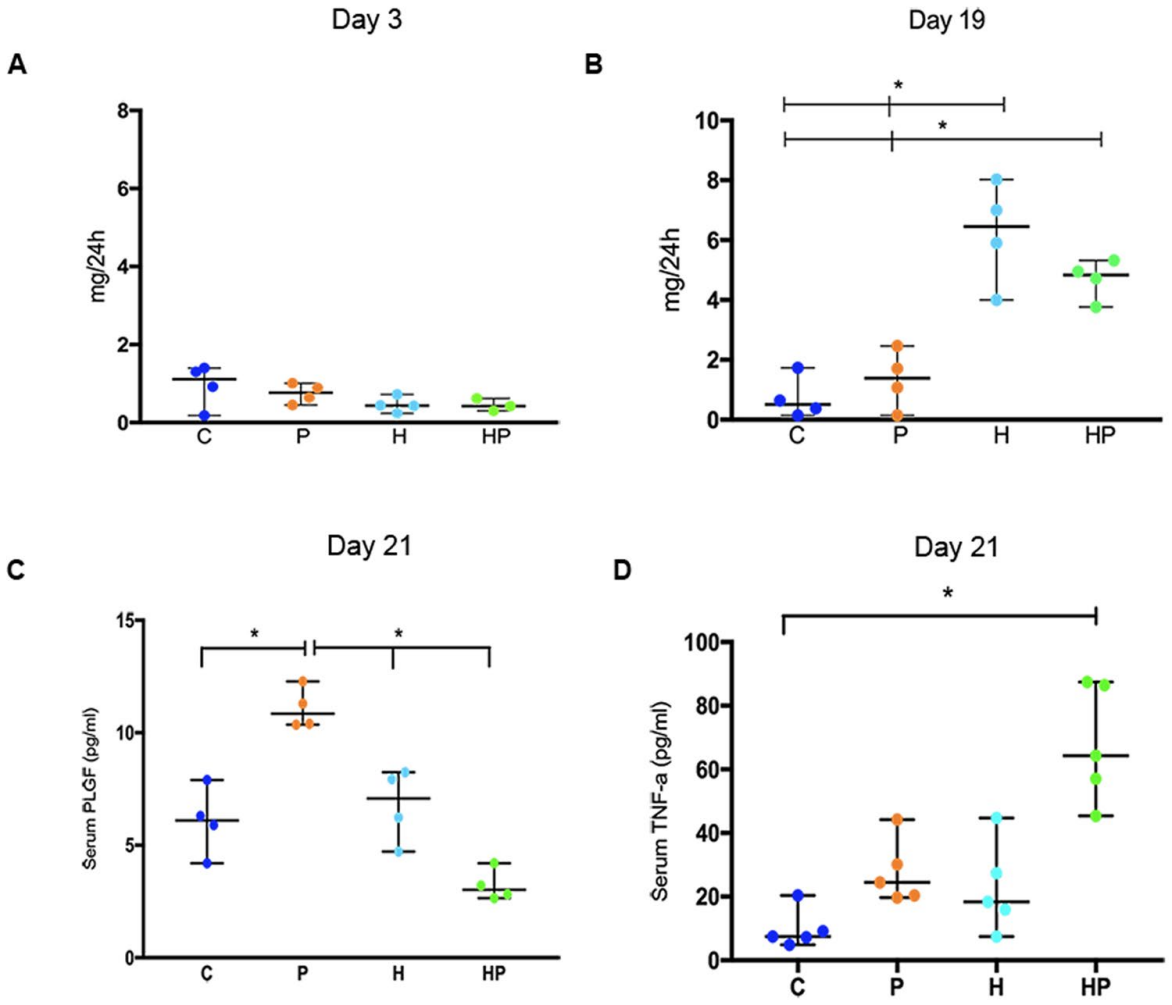
Biochemical markers. Urine albumin (**A**,**B**) and serum PLGF (**C**) and TNF-α (**D**) median levels with 95% CI in control (**C**), pregnant (P), hypertensive (H) and pregnant-hypertensive animals (HP) determined 3, 19 and 21 days after the clamping of the left renal artery. Levels indicated on the graph showing (*) were found to have statistically significant difference.

Mean value of albumin levels in urine on day 3 showed no significant difference among groups C, P, H and HP (F = 1.912; p = 0.185). On day 19, groups H and HP differed from groups C and P (F = 23.02; p < 0.0001) by displaying levels characteristic of proteinuria in human preeclampsia albumin levels in H and HP showed no statistically significant difference (Fig. 2A). On day 21, mean level of serum TNF-α was significantly increased in HP as compared with C group (H = 14.38; p = 0.0024), but no difference could not be ascertained among groups P, H and HP (Fig. 2B). Mean values of PLGF levels (determined on day 21) in the serum were not different among C, H and HP, but were found increased in P animals (F = 14.85; p = 0.0002) (Fig. 2C).

### EEG seizure pattern

**Figure 3:**
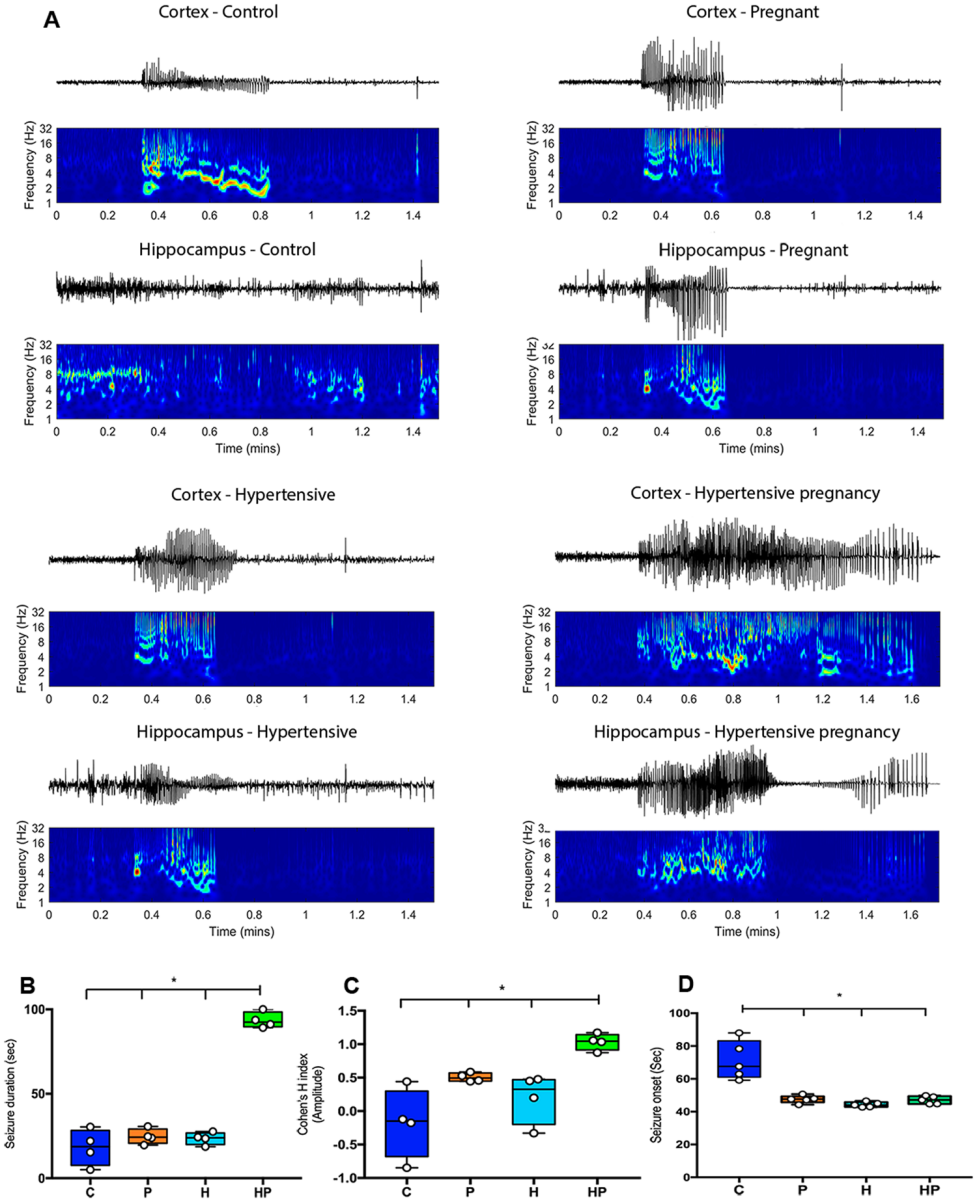
Electroencephalographic parameters *in vivo*. (**A**) One representative sampling of epileptiform activity in control (C), pregnant (P), hypertensive (H) and pregnant-hypertensive (HP) animals. EEGs coupled with graphs depicting seizure intensity throughout frequency spectrum with continuous wavelet transform using analytic Morse wavelet (hotter colors indicating higher intensities). Seizure duration (**B**), amplitude (**C**) and latency (**D**) in C, P, H and HP animals are represented with median. Levels indicated on the graph showing (*) were found to have statistically significant difference.

With the injection of PTZ, the first EEG signs of coming seizure start at about 46 seconds in groups P, H and HP which showed no difference in their respective latency. Group C, however, had a significantly increased latency (F = 20.55; p < 0.0001) of about as much as 50% as compared with their counterparts (Fig. 3D). On the other hand, the amplitude and duration of EEG signals in group HP were significantly increased (F = 9.56; p = 0.0017 and F = 152.7; p < 0.0001, respectively) as compared with the other groups which showed no differences among them (Fig. 3B,C). This findings were compatible with differences in behavior during seizure. All animals went through whisker trembling, sudden behavioral arrest, facial and neck jerking, clonic, tonic and tonic-clonic seizure. However, the higher stages in HP animals were dramatically more intense as compared with animals in the other groups with C animals experiencing seizure episodes only lightly.

### Seizure power derived from EEG

**Figure 4:**
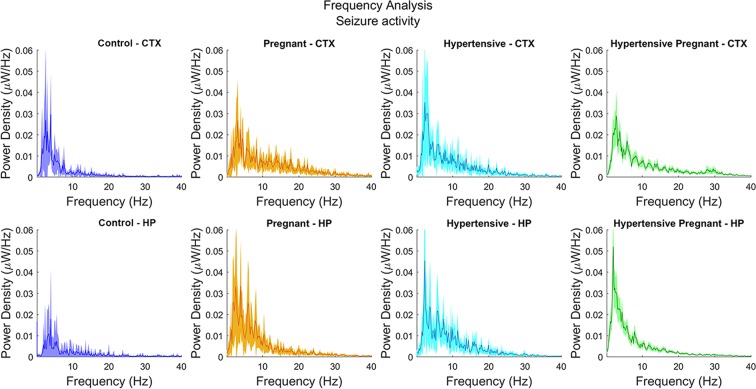
Seizure intensity by frequency. Normalized mean values of seizure power density (spiked line in darker shade) in the cortex (CTX) and hippocampus (HP) throughout the frequency spectrum in control, pregnant, hypertensive and pregnant-hypertensive groups with 95%-confidence interval (CI) represented by lighter shade. Notice the narrowest CI in pregnant-hypertensive group.

Although mean rates of power density plotted along the frequency spectrum had similar shape for all groups, HP had a narrower confidence interval (CI) as compared with the remaining groups (Fig. 4).

### *In vitro* seizure-like events

**Figure 5:**
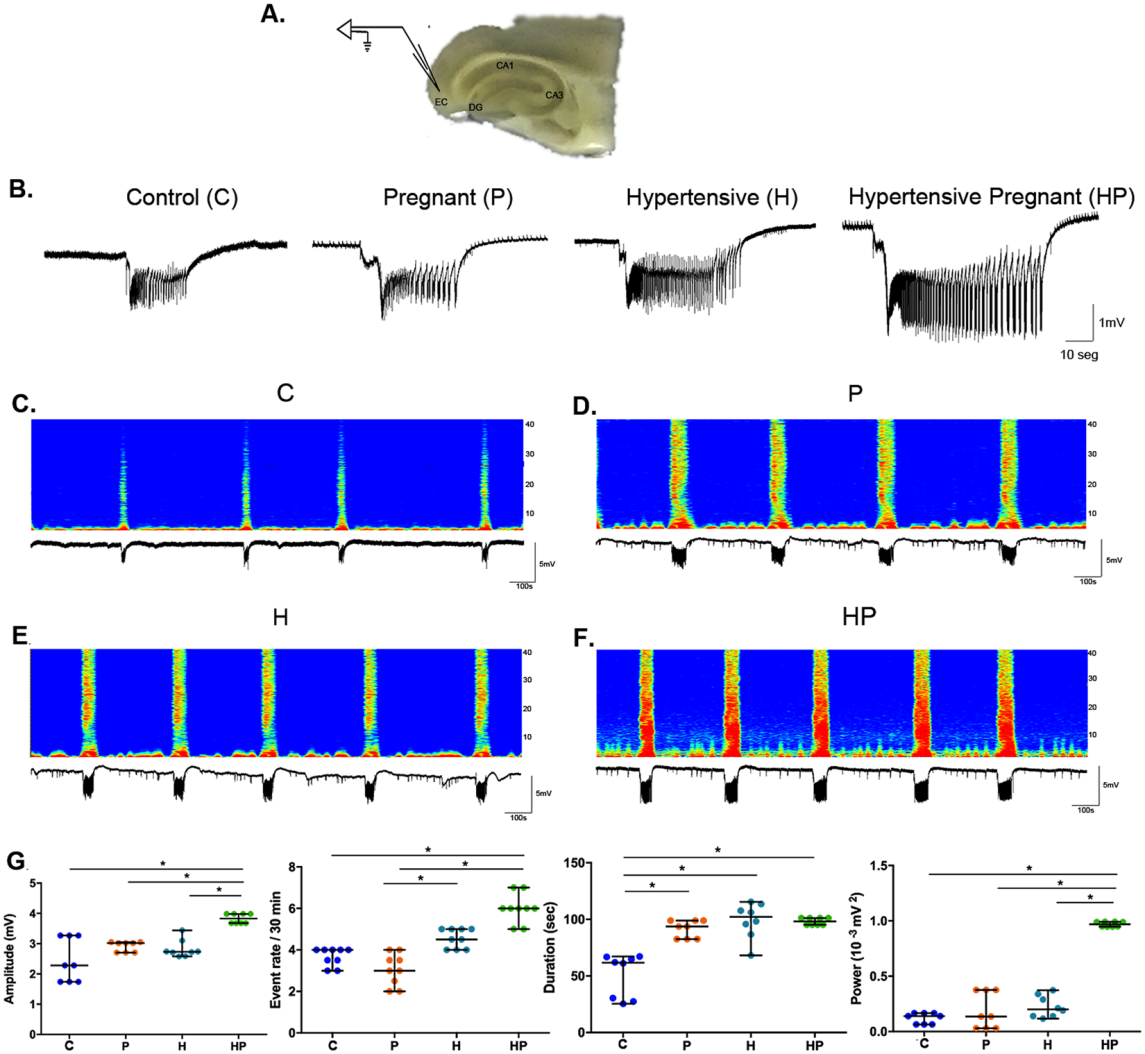
Electrophysiologic activity *in vitro*. Location of electrode in the entorhinal cortex (layer III-IV) for *in vitro* study on hippocampal section. (**A**) Representative signal recordings of seizure-like events in a slice from a control (C), pregnant (P), hypertensive (H) and pregnant-hypertensive (HP) animal (**B**) with corresponding graphical analyses of seizure magnitude throughout the frequency spectrum where hotter colors indicate greater magnitude. (**C**–**F**) Analysis of amplitude, rate, duration and power of seizure-like event in 3 slices of each animal in C, P, H and HP groups shown with median value represented on CI (95%). (**G**) Levels indicated on the graph showing (*) were found to have statistically significant difference.

Mean values of amplitude and power obtained from hippocampal slices were significantly increased in group HP as compared with those in C, P and H (F = 16.82; p < 0.0001 and H = 18.81; p = 0.0003, respectively) (Fig. 5G).

Duration was shorter in C animals as compared with those in groups P, H and HP (H = 19.94; p = 0.0002), among which no significant difference was found (Fig. 5G).

The mean number of seizure-like events in 30 minutes was not significantly different between groups HP and H whereas HP rate was increased as compared with those for P and C (H = 28.30; p < 0.0001). Furthermore, H rate was increased as compared with those in P (Fig. 5G).

### Immunochemistry

**Figure 6:**
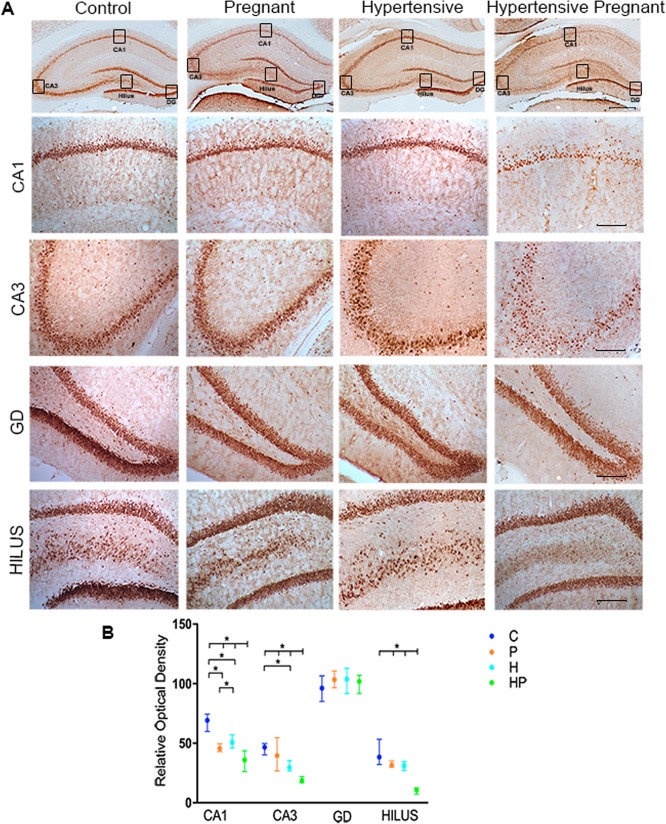
NeuN immunohistochemistry. Representative images of hippocampal sections from control (C), pregnant (P), hypertensive (H) and pregnant-hypertensive (HP) groups. Note the lighter staining of CA1, CA3 and Hilus in section of pregnant-hypertensive individual. Scale bar for the first row of images represents 500 μm and scale bars for subsequent rows 200 μm. (**A**) Analysis of optical density in CA1, CA3 Hilus and Dentate Gyrus of hippocampal sections with group median values indicated on CI (95%). (**B**) Levels indicated on the graph showing (*) were found to have statistically significant difference.

NeuN immunohistochemistry showed that mean values of relative density of neurons nuclei in CA1, CA3 and Hilus in HP animals were decreased as compared with C, P and H (H = 42.38; p < 0.0001 for CA1; H = 40.52; p < 0.0001 for CA2; and H = 43.32; p < 0.0001 for Hilus), but no difference was ascertained in Dentate Gyrus (GD) between groups C, P, H and HP (Fig. 6A,B).

**Figure 7:**
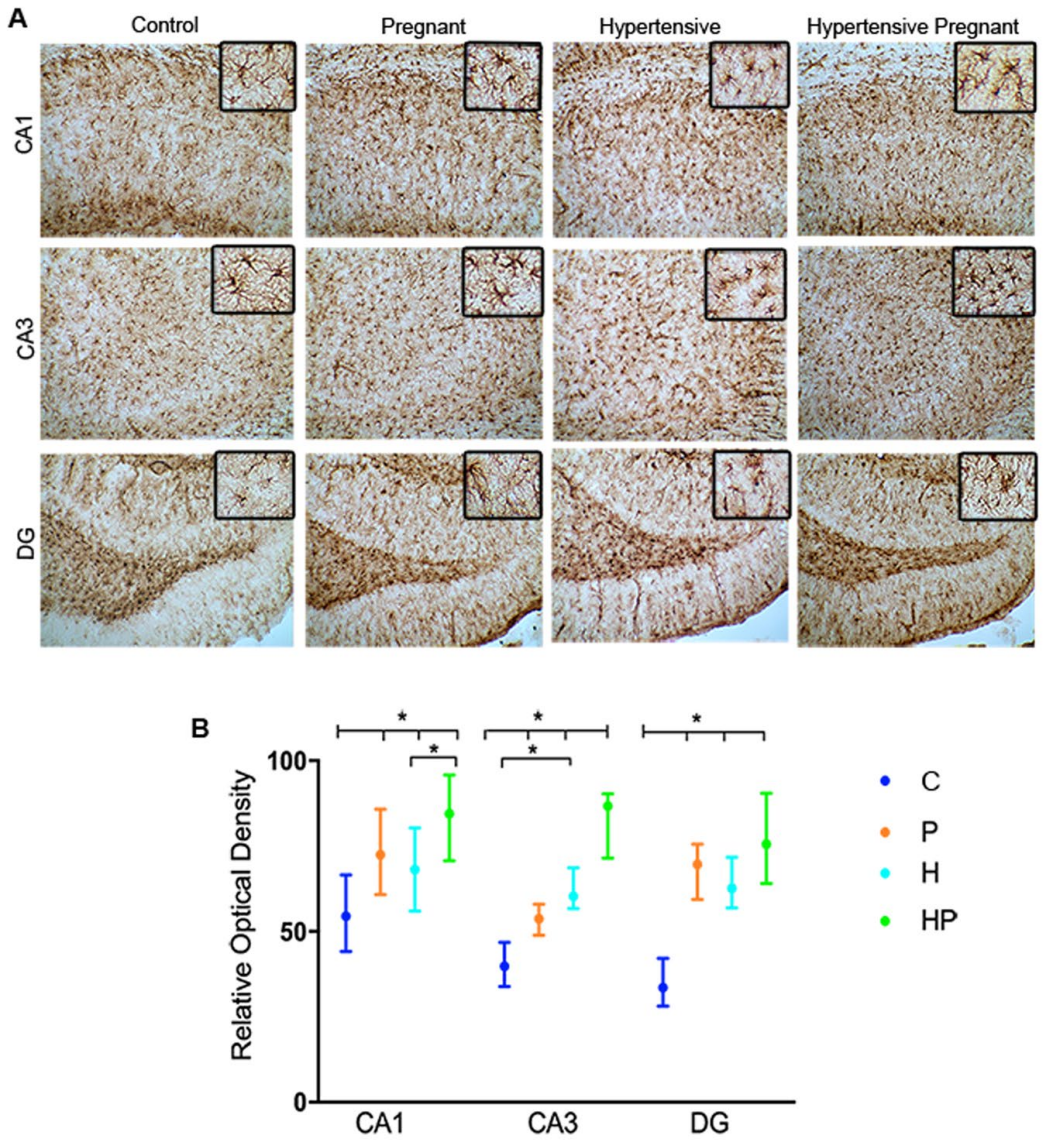
GFAP immunohistochemistry. Images (scale bar 50 μm) with enlarged area (scale bar 200 μm) of hippocampal sections from control (C), pregnant (P), hypertensive (H) and pregnant-hypertensive (HP) groups. Note that enlarged areas of HP individual contain more astrocytes than counterparts. (**A**) Analysis of optical density in CA1, CA3 and Dentate Gyrus of hippocampal sections with group median values indicated on CI (95%). (**B**) Levels indicated on the graph showing (*) were found to have statistically significant difference.

GFAP immunohistochemistry showed that HP animals had increased relative density of astrocytes indicating harder neuronal scarring in CA1, CA3 and GD of these individuals as compared with densities in C, P and H (animals (F = 11.46; p < 0.0001 for CA1; F = 48.77; p < 0.0001 for CA3 and F = 30.01; p < 0.0001 for GD) (Fig. 7A,B).

### Weight at birth

**Figure 8:**
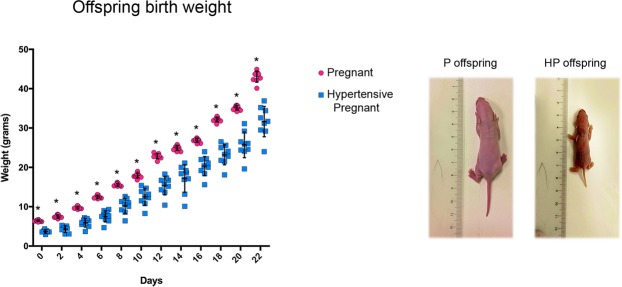
Offspring development. Weight follow-up of 22 days of pregnant (P) and pregnant-hypertensive (HP) offspring with individual values in the group represented in dispersion graph. Mean values are shown on standard-deviation long vertical bars. Images show 5-day pups from P and HP offsprings. Levels indicated on the graph showing (*) were found to have statistically significant difference.

On delivery day, mean weight of HP offspring was significantly reduced as compared with that of P offspring (p < 0.0001). Differences in weight persisted during follow-up of 22 days after birth (Fig. 8).

## Discussion

The possible causes of preeclampsia (PE) and their relationship to chronic kidney disease (CKD) are far from being established^21^. The difficulty in elucidating the puzzle apparently lies on the fact that the main markers for PE and CKD, such as increased blood pressure with proteinuria, increased levels of the soluble fms-like tyrosine kinase (sFlt1)^22^, increased levels of inflammatory markers^23^ and reduced levels of vascular endothelium growth factor, are present in both pathologies^9,24^.

Moreover, pregnant women aged 35 and above are not regularly tested for CKD, which could help to identify possible clues of underlying renal dysfunction. Conversely, studies show that some pregnant women with PE and clear renal dysfunction have the two conditions resolved within weeks after delivery without sequels^25,26^ whereas others had their risk of developing kidney dysfunction increased^4^. Van Balen *et al*. found that from 775 primiparous PE women without previous history of hypertension, diabetes or kidney diseases 13.7% had to be monitored for kidney dysfunction one year or more postpartum and 1.4% were classified as having higher risk for kidney function deterioration^27^.

The clinical management of preeclampsia involves strategies developed to prevent the occurrence of seizures that, unfortunately, are not routinely accompanied by electroencephalographic recordings. These could help in the recognition of cerebral rhythm fluctuations, the occurrence of isolated discharges, the more accurate location of these changes and the cerebral circuitry involved. One possible way to overcome these challenges depends on the use of animal models that reproduce, as faithfully as possible, the main characteristics of the human syndrome.

The literature presents mainly three ways of inducing PE models, that is, causing inflammation, mimicking the depletion of VEGF and PLGF or restricting blood flow to the placenta. Among the inflammatory models are those proposed by Faas *et al*.^16^ LaMarca *et al*.^18^ and Orshal and Khalil^17^, who infused lipopolysaccharides (LPS), TNF-α and interleukin-6 (IL-6), respectively. In their reports, however, arterial blood pressure levels (127–146 mm Hg) did not reach critical values compatible with those described for the human condition, which could be a good indicator that the inflammatory process per se was not able to reproduce the preeclamptic picture. The VEGF and PLGF depletion PE model reported by Maynard *et al*.^28^ was obtained by injecting adenovirus expressing sFlt-1 in the tail vein of rats, a method expected to inactivate endogenous VEGF and PLGF impairing the placental growth. In these circumstances, these authors were able to detect the occurrence of proteinuria, glomerular endotheliosis and high diastolic blood pressure, adding important elements for the physiopathology of PE. To our knowledge, however, these authors did not report changes in seizures susceptibility in their model. The third way of inducing a PE model has been proposed by Fushima *et al*.^29^, who used three different possibilities to restrict the blood supply to the placenta by clipping: (a) both ovarian arteries, (b) both uterine arteries, (c) both ovarian arteries plus both uterine arteries. Regardless of the clipping alternatives, max BP achieved was 124 ± 5.5 mmHg in experimental against 96.8 ± 1.9 mmHg in control animals, proteinuria was observed only when clipping uterine arteries, and no significant difference of serum sFlt-1 levels was obtained among the groups studied.

An interesting genetic engineering experiment in mouse achieved a SBP increase similar to that occurring in preeclamptic women at around the beginning of the sixth pregnancy month^30^. Takimoto *et al*. Mated transgenic males expressing renin to transgenic females expressing angiotensinogen, resulting in pregnant individuals that exhibited continuous rise in SBP from gestational day 14 until delivery with SBP rates reaching over 155 mmHg, which dropped to normal levels within 3 days after offspring birth^30^. It seems that the authors intended to mimic the deleterious influence of the placenta in PE with the entering of renin produced by the maturing fetus into the mother’s blood stream. The fetal renin working on maternal angiotensinogen causing a SBP rise that better reproduced the characteristic evolution of PE as compared with those achieved in the models previously described. Because the main focus of the authors was on gestational hypertension and its deleterious effect on the heart and kidney structures, no information on sFlt-1, PLGF, VEGF and TNF-α was reported^30^.

The 2K-1C model presently reported shows the characteristic preeclamptic SBP evolution combined with the additional markers of PE including proteinuria, increased TNF-α and reduced PLGF serum levels, reduced weight of offspring at birth and increased seizure susceptibility in regular *Wistar* rats without either employing genetic engineering, administration of any drugs or agents or surgical intervention after presumed gestational day one.

The condition more frequently reported to be associated with PE is glomerular kidney disease (GKD)^25^, whose hallmark is proteinuria thought to be the caused by an inflammatory condition^5,7^. In PE, it seems to be triggered by anti-angiogenic factors arising from mechanisms associated with a placenta that fails to develop properly^31^. In the present study, both H and HP groups showed increased levels of albumin in urine in late pregnancy (day 19).

Another finding common to chronic kidney disease (CKD) and PE is higher levels of soluble fms-like tyrosine kinase-1 (sFlt-1)^9,22^. In CKD studies, sFlt-1 is associated with endothelial disfunction and is believed to impair vascular regeneration by sequestrating VEGF^22^. Similarly in PE, sFlt-1 is reported to suppress free vascular growth factor (VEGF) and placental growth factor (PLGF) causing restricted fetal growth^32,33^. In the present study, hypertensive animals (group HP) showed underdeveloped fetuses and lower serum concentrations of PLGF, consistent with higher levels of sFlt-1, findings absent in normotensive pregnant counterparts (group P).

BP behavior commonly determines PE diagnoses with its hallmark (140/90 mmHg) appearing only around the beginning of the last gestational trimester^5–7^. In the attempt to establish an early warning of PE, some authors suggested that elevated levels of TNF-α would fit the role, as studies in pregnant women showed that PE group displayed increased levels of serum TNF-α, as compared with controls^6,34,35^. More than a marker, TNF-α is a pro-inflammatory factor also occurring in CKD and has been reported to facilitate seizures when in higher concentrations^36,37^. In the present 2K-1C model, TNF-α levels, determined on experimental day 21 for convenience, was increased in HP as compared with C animals. Although the eventual occurrence of spontaneous seizure or death, as reported in humans, was not observed, the susceptibility to fatal seizure was assessed by injecting pentylenetetrazol (PTZ), a GABA_A_ antagonist^38^.

The excitatory mechanism involving TNF-α, produces the endocytosis of inhibitory gamma-amino-butyric acid (GABA_A_) receptors and exocytosis of excitatory AMPA receptors in hippocampal pyramidal cells, making them more excitable^36^. The injection of PTZ was intended to block available GABA_A_ receptors in order to determine to what extent the animals’ endogenous inhibitory resources could fight seizure. A statistically significant decrease in latency, larger amplitude and extended seizure duration was found in HP animals as compared with C controls, consistent with HP group increased levels of serum TNF-α, confirming the role of this deleterious cytokine as a predictor of PE severity and consequent susceptibility to seizure.

The increased levels of serum TNF-α in P and H groups were found to have no statistical significance when compared with C, but significant decreased latency was found in P and H as compared with C. This discrepancy can well result from factors other than serum TNF-α. Johnson *et al*. administering PTZ in *Sprague Dawley* rats concluded from Western blots that there was a downregulation of GABA_A_ receptor in pregnant animals as compared with non-pregnant ones. In pregnant animals, the expressions of cortical GABA_A_R-δ and hippocampal GABA_A_R-γ2 subunits were found decreased. This reflected on decreased EEG latency in pregnant as compared with non-pregnant individuals^39^. Furthermore, Cipolla *et al*. showed that hippocampal neurons developed increased excitability when hippocampal slices were cultured in serum of pregnant rats, but not in serum of non-pregnant, despite both sera having similar levels of TNF-α content. These authors hypothesized that such increased excitability was linked to a factor, again other than TNF-α, that existed in the serum of pregnant rats, which did not in serum of non-pregnant ones^37^.

In the present study, H and HP groups showed increased susceptibility to seizure denoted by decreased latency as compared with C counterpart. The finding by Scorza *et al*. seems to sheds some light on how hypertension facilitates seizure episodes. These authors related hypertensive condition to micro infarctions in the capillary arrays of vascular supply to the brain, known to be a potential adjuvant of seizures^40^. Additionally, Deyn and McDonald observed that patients with severe renal insufficiency had levels of guanidinosuccinic acid, creatinine, guanidine and methylguanidine in their blood and cerebrospinal fluid as much as ten times higher than normal^41^. The experiments of the authors were suggestive that these substances inhibit the responses to inhibitory neurotransmitters GABA and glycine by blocking the chloride channels^41^, thus supporting the increased susceptibility to seizure of hypertensive individuals showing advanced renal function deterioration as is the case of H and HP animals denoted by their levels of SBP and proteinuria.

The magnitude of neuronal activity during seizure was analyzed by mathematically treating the EEG signals by bands of frequency (Fig. 4). The maximum and minimum values of a representative total measure of neuronal activity, named power density, cover a statistical range of 95% of a population assumed to be represented by the mean of EEG signals average values, and their variation, acquired in a given range of frequency, for each animal in the study group.

The behavior of mean values along the spectrum of frequency did not differ significantly among groups, but HP group showed a significantly narrower interval of minimum and maximum of power density for a given frequency. The statistical meaning of this finding is that the seizure condition was more evenly distributed in HP group since the assumed population concentrated heavily around the mean. This statistical concept is reflected on the significantly larger number of animal (not shown) that died during seizure in HP group.

Amburgey *et al*. reported that plasma from normotensive pregnant women as well as preeclamptic women increased the permeability of rat’s blood-brain barrier (BBB) in 6.5- and 18-fold, respectively^42^. Assuming that BBB permeability could possibly be a confounding variable, the present research work included *in vitro* experiments in order to determine a possible influence of BBB permeability on the amount of PTZ reaching brain tissue of pregnant and non-pregnant animals.

The *in vitro* seizure events were obtained by exposing brain slices to 4-aminopyridine (4-AP). These events were definitely more powerful, had larger amplitude and were more frequent in 30-minute observation in HP group as compared with the other study groups (Fig. 5G), keeping consistence with respective power density by frequency analysis (Fig. 4).

Concerning seizure duration *in vitro*, P, H and HP groups did not differ significantly with durations of 91.50 ± 2.71 s, 98.99 ± 5.53 s and 98.18 ± 1.08 s, respectively and were significantly longer than duration in C (50.69 ± 6.76 sec), whereas seizure duration *in vivo* did not differ among C, P and H, but was longer in HP (Fig. 3B). These findings seems to imply that the association of pregnancy and hypertension factors increased BBB permeability letting a greater amount of PTZ reach brain tissue of HP animals.

The distinctive intensity of seizure in HP animals, reflected on distinctive loss of neurons, was ascertained by immunohistochemistry. A remarkable change in staining density occurred along the characteristic hippocampal staining lines, particularly in regions CA1, CA3 and Hilus as shown by NeuN immunohistochemistry (Fig. 6A,B). Similar findings have also been reported in epilepsy studies^43^. Accordingly, astrocyte proliferation, which follows neuronal loss, also confirms the seizure intensity^44^. This is shown in Fig. 7A,B with controls exhibiting the least density of GFAP staining followed by P, H and HP in order of increasing staining density.

In conclusion, clamping one of the renal arteries to 0.15 mm was an effective and reliable approach for producing characteristic human preeclampsia features, i.e., high blood pressure, proteinuria, increased TNF-α and reduced PLGF serum levels, reduced weight of offspring at birth and increased seizure susceptibility *in vivo* and *in vitro*.

Although the present 2K-1C model was not able to produce spontaneous seizure and ensuing death, as expected in untreated human pre-eclampsia, the present model showed animals with severe neurological injury and increased susceptibility to seizures.

Previous papers have implicated the significant contribution of placental alterations as key factors in the physiopathology of PE^2,28,29,32^. Unfortunately, our work did not focus on the study of placental alterations in this model and, accordingly, new experimental studies directed to these alterations are being planned by our group.

As a last note, the authors would like to make it clear that they are well aware that addressing complex pathologies with animal modeling is not an easy task, mainly when the etiological agent or mechanism remains elusive as it is with preeclampsia. It is not unusual the instance when different animal models can represent only one facet of the disease itself – the etiological mechanisms, the underlying pathophysiologic processes, the clinical manifestations and the alternative treatments. There may be not an ideal model that presents all information we need for comprehending human condition. And despite this entire complex situation, the experimental models have been instrumental in improving our knowledge, monitoring and control of human diseases to more adequate levels.

All previous models mentioned in this study have their own value by addressing very useful aspect for understanding the underlying mechanisms associated with PE, such as changes in the inflammatory cascade and/or ischemic placental changes. But in these models, the changes in systolic blood pressure (SBP) are minimal, which contrasts with the clinical findings, in which increased SBP is an alert for the possibility of PE.

Our intention was to represent the main clinical features that best characterize preeclampsia, that is, arterial hypertension during pregnancy associated with increased susceptibility to convulsive seizures, biochemical changes related to renal dysfunction, and offspring with low birth weight. Certainly the present adaptation of the 2K-1C model raises a renal issue that should be taken into consideration in further use of it.

## Methods

All animal handling and experimental procedures complied with the guidelines for animal care and use of laboratory animal and received the approval from the Board for Ethics in the Use of Animal (CEUA – Comissão de Ética no uso de animais) an institutional ethics committee of the Federal University of São Paulo (UNIFESP) under number 8821020715.

### Animals

A total of 56 female *Wistar* rats weighing 180 to 220 g were randomly allocated into 4 groups of 14 animals each: control (C), pregnant (P), hypertensive (H) and hypertensive-pregnant (HP) groups. The animals were kept (four per cage) at 21 °C in light/dark cycle of 12/12 h with free access to food and water. Twenty-eight animals (groups P and HP) were mated during the estrous phase and the presence of spermatozoid in the vaginal smear was established as experimental day 0 of the pregnancy period. These pregnant rats were then divided into 2 different groups: one underwent hypertension induction by the clamping of the left renal artery (hypertensive-pregnant group, HP) and the other group underwent sham surgery (P) in order to be control for the hypertensive condition in pregnancy. The other 28 female *Wistar* rats not undergoing mating were also divided into 2 other groups: one group had the left renal artery clamped (hypertensive group, H) and the other was taken as general control to both experimental variables, i.e., pregnancy and hypertension (Control group, C).

### Hypertension induction (artery clamping)

Groups HP and H underwent surgical procedure for induction of hypertension. In pregnant female rats it was performed soon after the detection of spermatozoid in their vaginal smear making pregnancy day 0 also surgical day 0 (baseline for hypertension induction). Surgical procedure was based on a previous paper^45^. Animals were anesthetized with Xylazine 10 mg/kg i.p. (Anasedan^®^) and Ketamine 100 mg/kg i.p. (Dopalen^®^). The left renal artery (LRA) was accessed through dorsal incision and a flat U-shaped silver strip (2 cm × 0.2 mm) was clamped on it to the aperture of 0.15 mm, regular suturing was performed and animals were housed and left for recovery. Groups P and C were submitted to sham surgery, i.e, the left renal artery was accessed but not clamped.

### EEG - Electrodes implantation

During the surgical procedure for hypertension induction but previously to renal artery clamping, 4 rats from each group were placed in a stereotaxic apparatus. A pair of electrodes (NiCr 150 μm DIA) aimed at the left hippocampal CA1 area (AP 3.8, LL 2.5 and DV 2.8; Paxinos and Watson^46^). A second pair of electrodes (100 μm DIA) was implanted in the left and right frontoparietal cortex, posteriorly and close to the bregmatic suture. All four electrodes were plugged into a micro-connector fixed to the animal’s skull with polymerizing acrylic resin.

### Blood pressure recording

Systolic blood pressure (SPB) was taken with a tail-cuff BP-2000 Blood Pressure Analysis System, *Visitech Systems*. All animals were accustomed to measuring procedures by being subjected to them for 5 consecutive days before day 0. SBP was assessed on days 2, 8, 14 and 19 and consisted of taking SBP 10 times with interval of 5 seconds between each take and calculating the mean value of the series.

### Urine collection for proteinuria determination

Four animals from each groups were kept in metabolic cages for 24 h on days 3 and 19 with free access to food and water in order to collect material for proteinuria analyses.

### PLGF, TNF-α and albumin determination

Immediately after delivery or experimental day 21, 4 animals from each group were decapitated and blood was collected and centrifuged at 4 °C for serum collection and storage at −80 °C. PLGF and TNFα concentrations were determined using commercial solid-phase sandwich enzyme-linked immunosorbent assay (ELISA) kits (Elabscience Biotechnology Co.Ltd, China). Albumin concentration was determined using ELISA kit from Bethyl Laboratories (Montgomey, TX, USA) with readings performed in multimode plate reader (PerkinElmer, VICTOR X3, Singapore).

### Seizure induction

In order to induced seizure, pentylenetetrazol (PTZ) (P6500-Sigma-Aldrich, St. Louis, MO - USA) in pyrogen-free saline solution at 0.9% was i.p. injected into 11 animals from each groups totalizing 44 animals at the dose of 50 mg/kg 15 and 18 days following the surgical procedures.

### *In vivo* electrographic recordings

EEG was recorded with animals in a *Faraday* cage. Signals were amplified (Grass instruments model RPS 107), digitalized (CED micro 1401-3, Cambridge, United Kingdom) and acquired using *software* Spike 2 v6.09^®^. EEG monitoring and data acquisition was performed for 5 hours. Seizure duration (period of time from onset of generalized seizure to the beginning of postictal depression), amplitude (variation from baseline signal to generalized seizure and decline to postictal onset using Cohen’s H-index)^47^ and latency (time between PTZ injection and first seizure spike) were assessed from EEG recordings using homemade scripts in MATLAB 2017^®^.

For every group, the electroencephalographic recordings were segmented into stretches of 5 seconds with 75% overlap and Fourier transform was used in order to obtain the power spectrum in the frequency domain. Mean values of power spectrum over time are shown with its confident interval CI (95%) (Fig. 4).

### *In vitro* hippocampal recordings

Immediately after delivery or experimental day 21, 3 animals from each group were anesthetized with 1% isoflurane in 70% N2O and 30% O2, decapitated, and had their brains immediately removed and hemisected, and horizontal slices were obtained containing the hippocampal formation, temporal, perirhinal and entorhinal cortex and a total of 9 slices per group were taken for *in vitro* study. Slices were prepared as described by Guimarães Marques *et al*.^48^ with brain tissue sliced in carbogenated ice-cold artificial cerebrospinal fluid (aCSF) at 4° ± 0.5 °C temperature composed by (in mM) NaCl 129, NaHCO3 21, KCl 3, CaCl2 1.6, MgSO4 1.8, NaH2PO4 1.25, and glucose 10, saturated with 95% O2 and 5% CO2. Slices were cut at thickness of 400 µm using a vibratome (LEICA VT 1200 S). Then, slices were immediately transferred to an interface chamber perfused with aCSF at 36 ± 0.5 °C (flow rate: 1.5–2.0 ml/min, pH 7.4, osmolarity: 295–300 mOsmol/L) and after allowing brain slices to recover for 2–3 hours, the electrophysiological recordings were started. Extracellular recordings were performed using glass pipette filled with 154 mM NaCl, with tip diameter 2–3 mM, resistance 2 to 4 MΩ, positioned in the layer III-IV of the entorhinal cortex. Epileptiform activity type seizure-like events (SLEs) were induced by 4-aminopyridine (4-AP), (100 mM, Sigma Aldrich). Signals were amplified using a custom-made amplifier equipped with capacitance and offset potential compensation, filtered at 3 KHz, digitized on-line (CED-1401, Cambridge, United Kingdom) and stored for off-line analysis using Spike 2 v6.09 (CED-1401, Cambridge, United Kingdom). Event rate (events per minute), duration (from onset up to two-thirds recovery of the shift field potential), amplitude (peak-to-peak) and power (band frequency of 73–293 Hz)^49^ of SLEs were measured using homemade scripts (MATLAB 2017^®^) for 30 minutes after epileptiform activity becoming constant regarding event rate and amplitude.

### NeuN and GFAP Immunohistochemistry

Immediately after delivery or experimental day 21, 4 animals from each group provided hippocampal sections for neuronal nuclei (NeuN) and glial fibrillary acidic protein (GFAP) antibody staining^50^ (1:50 – Chemical – Milipore – AB377 with IgG goat anti-mouse 1:200 AB6789 and 1:5000 – Abcam – AB7260 with igG goat anti-rabbit 1:200 AB6721, respectively) in order to assess neuronal loss and neurological scarring. Assessment was performed through optical density^50^.

### Offspring birth weight

As a side observation, a total of 9 offspring individuals from each P and HP group were weighed on delivery day (experimental day 21) and followed for 22 days with weight take on every two days in order to determine any difference in growing pattern between offsprings of these groups.

### Statistical analysis

Shapiro-Wilk’s normality test was used to verify normal distribution of samples origin. ANOVA and Tukey’s *post-hoc* test were used to ascertain statistically significant difference among groups and identification of the pairwise significant differences, respectively. Alternatively to parametric statistical tools, Kruskal-Wallis test with Dunn’s *post-hoc* test were used for group differences. Level of statistical significance was set to 0.05. Confidence interval of 95% for median and mean were represented.

## Supplementary information

Supplementary information
