# Supplementary material for: Behavioral, electrophysiological and neuropathological characteristics of the occurrence of hypertension in pregnant rats
Source: Sci Rep. 2019 Mar 11;9:4051. doi: 10.1038/s41598-019-40969-w (PMC6412065; doi:10.1038/s41598-019-40969-w)

**Behavioral, electrophysiological and neuropathological characteristics of the occurrence of hypertension in pregnant rats**

Leandro F. Oliveira^1*^, Daniel J.L.L Pinheiro^1^, Laís D. Rodrigues**^1^**, Selvin Z. Reyes-Garcia^1,4^, Erika E. Nishi^2^, Milene S. Ormanji**^3^**, Jean Faber**^1^**, Esper A. Cavalheiro**^1^**.

^1^Department of Neurology and Neurosurgery, UNIFESP/EPM, Brazil

^2^Department of Physiology, UNIFESP/EPM, Brazil

^3^Department of Nephrology, UNIFESP/EPM, Brazil.

^4^Department of morphological science, Faculty of Medical Sciences, National Autonomous University of Honduras, Honduras.

*Corresponding author: [leandrof.neuropsicologia@gmail.com](mailto:leandrof.neuropsicologia@gmail.com)

**Supplementary material**

**Fig 9.** Hypertensive pregnant animal’s electroencephalographic (EEG) excerpts expanded in time. EEG from hypertensive pregnant animals show cortical (red) desynchronization (low amplitude) and hippocampal (blue) theta activity of about 7Hz characteristic of attentive wake. Calibration 1 second 50μV (**A** and **B**). Cortical and hippocampal generalized high-voltage spike wave and poly-spike wave of about 3 to 4Hz **(C** to **O)**. Cortical and hippocampal decreased voltage (postictal depression) **(P** to **R)**.


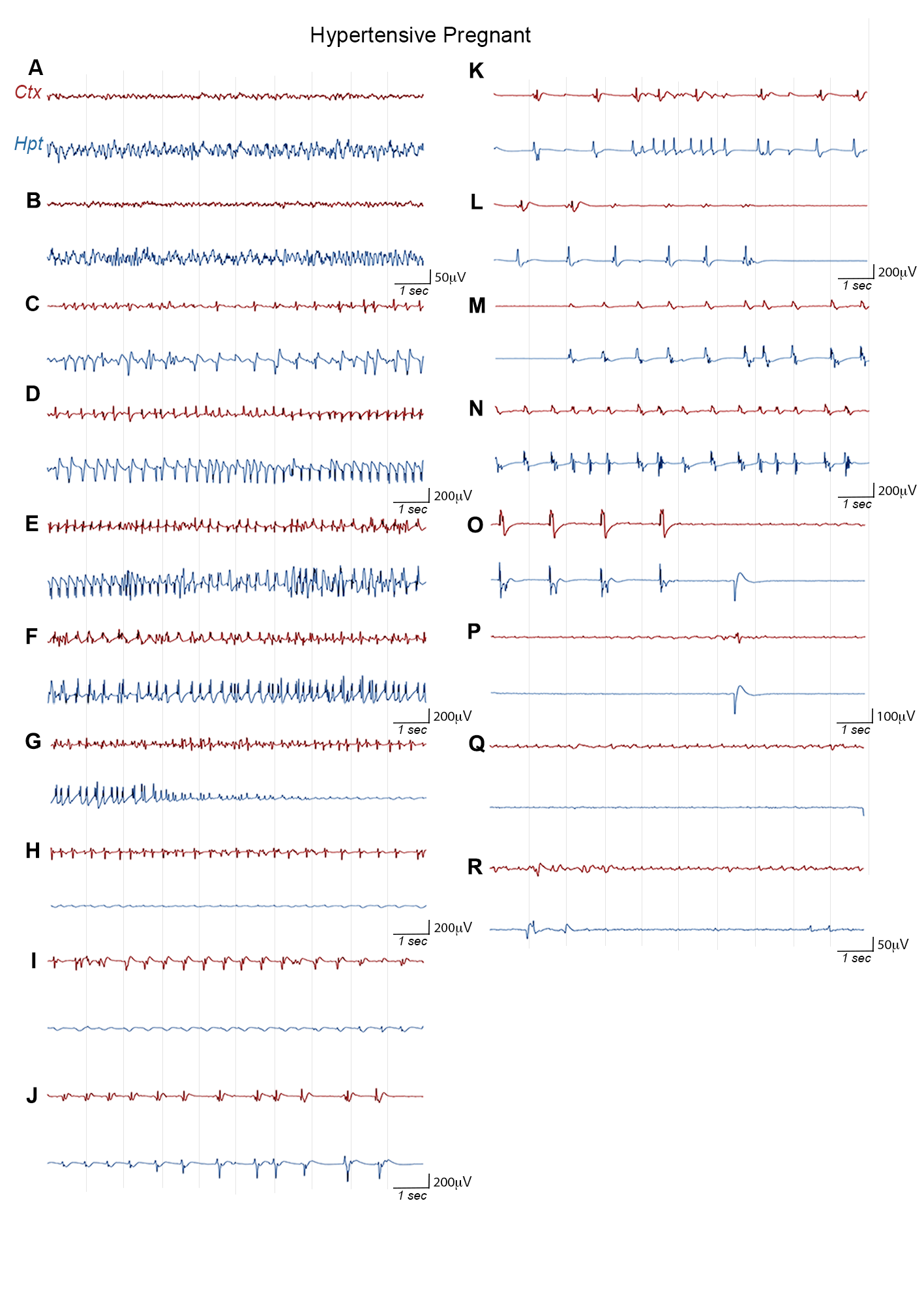


**Fig 10.** Hypertensive and pregnant animal’s electroencephalographic (EEG) excerpts expanded in time. EEG from hypertensive animals show cortical (blue) desynchronization (low amplitude) and hippocampal (red) theta activity of about 7Hz characteristic of attentive wake. Calibration 1 second 50μV **(A** to **D)**. Cortical and hippocampal generalized high-voltage spike wave and poly-spike wave of about 3Hz **(E** to **G)**. Decreased-voltage cortical signals (postictal depression) and paroxysmal epileptiform activity in the hippocampus **(H** to **L)**. EEG excerpts from pregnant animals show cortical desynchronization (low voltage and high frequency signals) and hippocampal theta activity of about 7Hz characteristic of attentive wake. Calibration 1 second 50μV **(A** to **D)**. Cortical and hippocampal generalized high-voltage spike wave and poly-spike wave of about 3Hz **(E** to **H)**. Cortical and hippocampal decreased voltage activity (postictal depression) **(I** to **L)**.


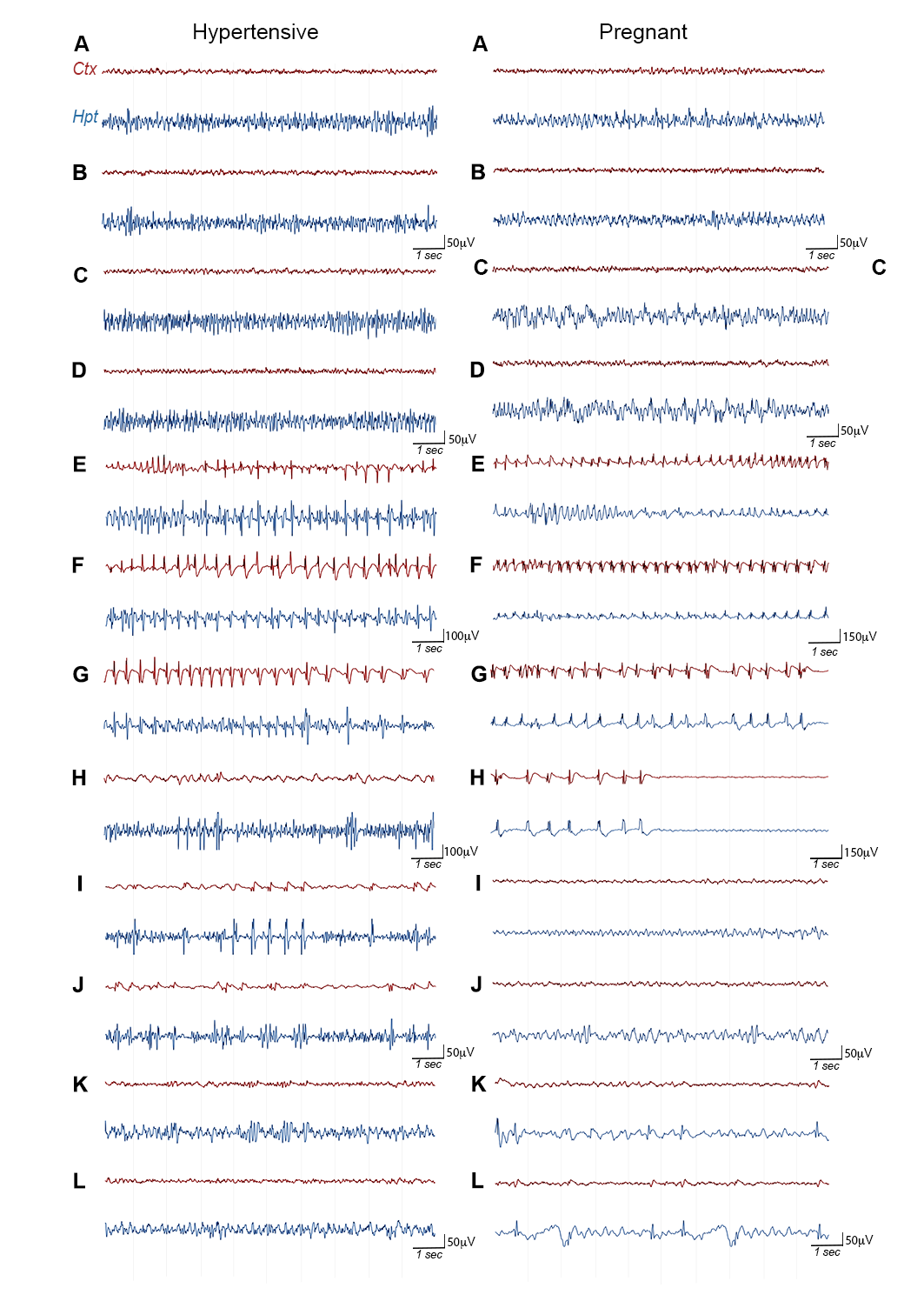


**Fig 11.** Control animal’s electroencephalographic (EEG) excerpts expanded in time. EEG from control animals show cortical desynchronization (low amplitude) and hippocampal theta activity of about 7Hz characteristic of attentive wake. Calibration 1 second 50μV **(A** to **C)**. Cortical and hippocampal generalized high-voltage spike wave and poly-spike wave of about 3Hz **(D** to **E)**. Decreased-voltage cortical signals (postictal depression) and paroxysmal epileptiform activity in the hippocampus **(F** to **H)**.


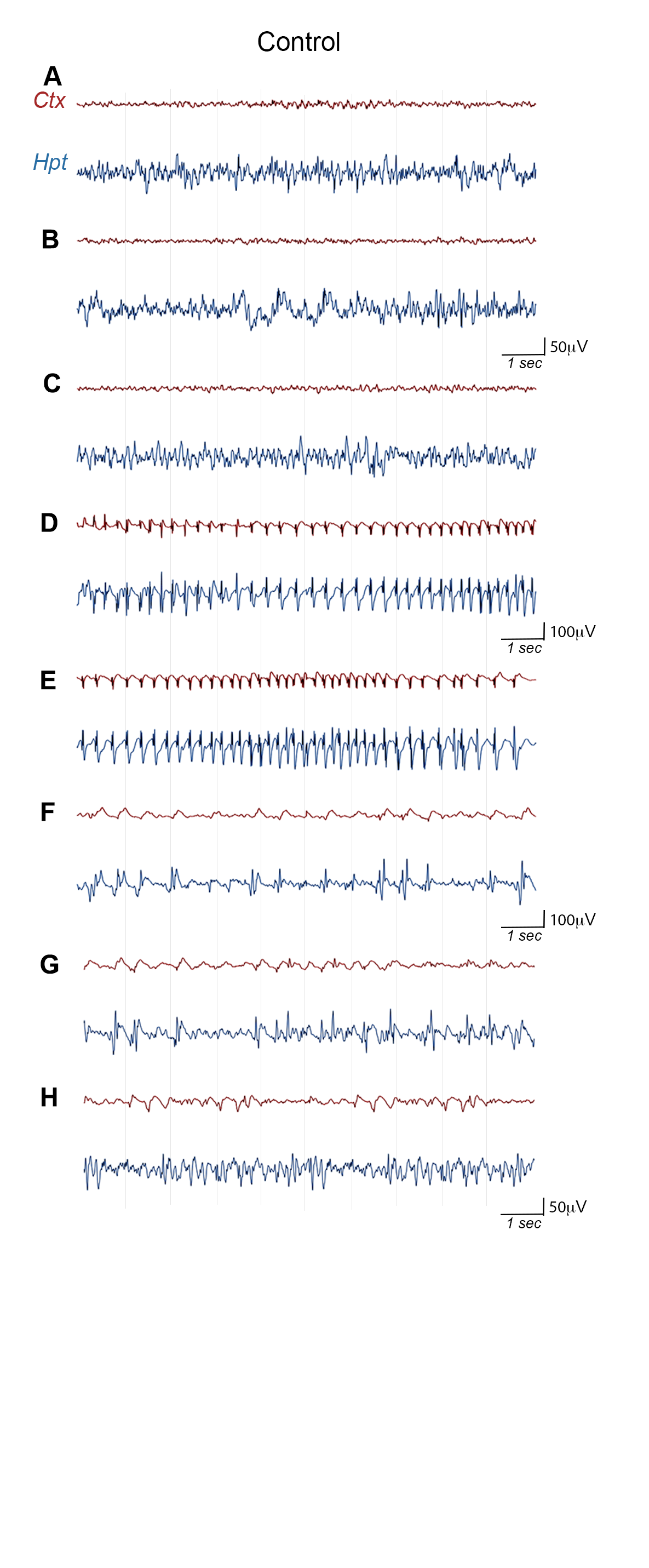


**Fig 12.** Statistical analysis of Systolic Blood Pressure (SBP). SBP levels on days 14 and 19 represented with mean values and CI (95%), shown significant differences (*p<0.05).


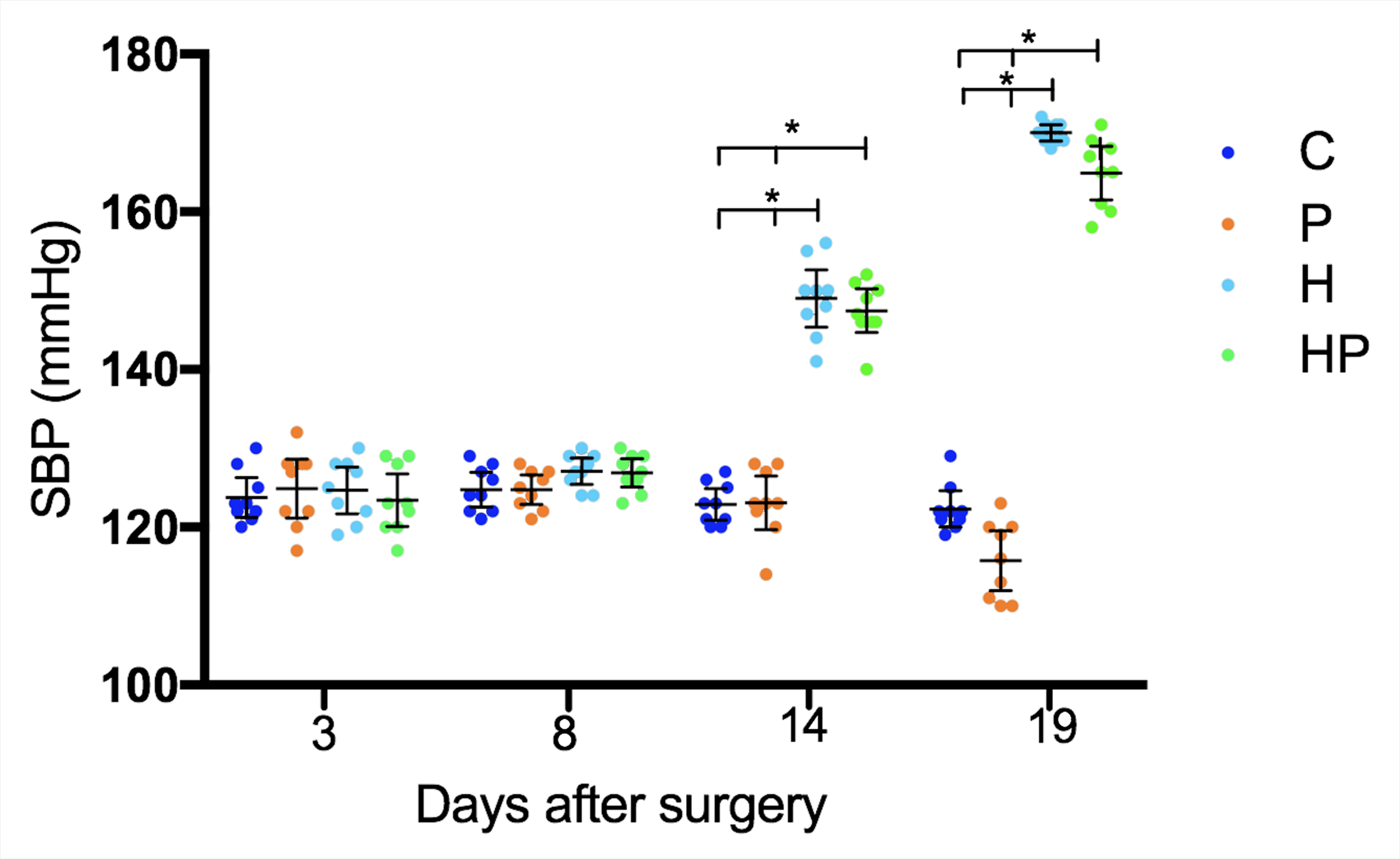

Supplement: Supplementary file 1 — Supplementary information [file 41598_2019_40969_MOESM1_ESM.docx]
